# Chemosensory adaptations of the mountain fly *Drosophila nigrosparsa* (Insecta: Diptera) through genomics’ and structural biology’s lenses

**DOI:** 10.1038/srep43770

**Published:** 2017-03-03

**Authors:** Francesco Cicconardi, Daniele Di Marino, Pier Paolo Olimpieri, Wolfgang Arthofer, Birgit C. Schlick-Steiner, Florian M. Steiner

**Affiliations:** 1 Institute of Ecology, University of Innsbruck, Technikerstr. 25, 6020 Innsbruck, Austria; 2 Department of Informatics, Institute of Computational Science, University of Italian Switzerland, Lugano, Switzerland; 3 Department of Physics, Sapienza University of Rome, Rome, Italy

## Abstract

Chemoreception is essential for survival. Some chemicals signal the presence of nutrients or toxins, others the proximity of mating partners, competitors, or predators. Chemical signal transduction has therefore been studied in multiple organisms. In *Drosophila* species, a number of odorant receptor genes and various other types of chemoreceptors were found. Three main gene families encode for membrane receptors and one for globular proteins that shuttle compounds with different degrees of affinity and specificity towards receptors. By sequencing the genome of *Drosophila nigrosparsa*, a habitat specialist restricted to montane/alpine environment, and combining genomics and structural biology techniques, we characterised odorant, gustatory, ionotropic receptors and odorant binding proteins, annotating 189 loci and modelling the protein structure of two ionotropic receptors and one odorant binding protein. We hypothesise that the *D. nigrosparsa* genome experienced gene loss and various evolutionary pressures (diversifying positive selection, relaxation, and pseudogenisation), as well as structural modification in the geometry and electrostatic potential of the two ionotropic receptor binding sites. We discuss possible trajectories in chemosensory adaptation processes, possibly enhancing compound affinity and mediating the evolution of more specialized food, and a fine-tuned mechanism of adaptation.

Chemosensory systems allow animals to orient themselves in a sea of chemical compounds. Some of these chemicals signal the presence of nutrients or toxins, others the proximity of mating partners, competitors, or predators. Signal intensity can be crucial: The quantity of sugar in a food source reflects its nutrient value, just like bitter compounds may reflect its toxicity, and the same stimuli can be attractive at low but aversive at high concentrations^1^,^2^. Being able to identify and quantify chemicals is crucial to survival and adaptation^1^,^2^.

Chemical signal transduction has been well studied and is generally similar in multiple organisms across the tree of life. From nematodes to crustaceans, insects, and vertebrates, chemical compounds are detected through interactions with specific receptors on the dendrites of both olfactory sensory neurons and gustatory receptor neurons^3^. The activation of these receptors induces a messenger cascade leading to ion channel activation and receptor neuron depolarization^4^. So far, four large gene families were discovered to mediate chemical responses from the environment, and further kinds of chemoreceptors likely await discovery^5^. Three of these gene families encode for membrane receptors (odorant, gustatory, and ionotropic receptors), while a fourth encodes for odorant binding proteins, globular proteins secreted by sensory neuron accessory cells^1^.

The three-dimensional arrangement of the insect odorant receptor (OR) family is still under debate since no clear structural information is available yet. Seven putative transmembrane helices (7-TMHs) were predicted^1^,^6^. In insect ORs, the amino-terminus is located toward the cytoplasm, and a ligand-gated ion channel function was inferred, which implies a signal transduction mechanism independent from G proteins^6^,^7^. Therefore, the odour response spectrum, the termination kinetics, and the level of spontaneous activity are hypothesized to be directly dependent on the receptor itself^4^,^8^, mediating both excitatory and inhibitory responses in the same cell^9^. The functional unit of the OR family consists of homomeric and heteromeric structures^6^ that involve the interaction of single odorant receptors with a co-receptor protein, as shown for Or22a and Orco^8^,^10^,^11^.

Compared with ORs, gustatory receptors (GRs) are a more divergent gene family and are generally more related to sweet, bitter, umami, and salt concentration taste^12^,^13^. They are just weakly related to ORs, with which they share the putative seven helices. Knowledge about GRs’ three-dimensional structure and their mechanisms of signal transduction is very poor^1^,^14^.

The ionotropic receptors (IRs) are related to ionotropic glutamate receptors (iGluRs), which mediate the excitatory transmission and are key to synaptic plasticity and to learning and memory processes^15^. Most IRs have two conserved domains: An extracellular 1^st^ half ligand-binding domain (S1) (*Lig_chan-Glu_bd*; Pfam: PF10613) at the amino-terminus followed by the ligand-gated ion channel domain (*Lig_chan*; Pfam: PF00060), which is composed of three transmembrane helices (TMHs), an ion channel pore (P), and the 2^nd^ half of the ligand-binding domain (S2). A third domain, the amino terminal domain (ATD), present in all iGluRs, can be present, depending on the gene and species, and does not share a strong homology signal with the iGluR sub-family^16^. Unlike other receptors, IRs confer response to many organic acids and amines, some of which play important behavioural roles, such as the induction of male courtship by food-related odour^17^.

The fourth gene family, the odorant binding proteins (OBPs), is a quite divergent family. It encodes for small globular and soluble proteins, which bind hydrophilic odorant compounds with different degrees of affinity and specificity to shuttle them to the underlying receptors, in form monomers and/or homodimers^18^. OBPs appear to be involved in odorant recognition, binding compounds, and in *Drosophila* are generally subdivided in six sub-families based on structural features, functional information, and phylogenetic relationships: *Classic, Minus-C, Plus-C, Dimer, ABPII,* and *CRLBP*^18^,^19^.

*Drosophila* species are saprophagous insects that feed and breed on a wide range of fermenting plant materials including fruits, flowers, slime fluxes, decaying bark, leaves, and stems, and on fungi^20^,^21^. Because of this, *Drosophila* species provide an excellent system to study how changes in the chemosensory repertoire are associated with behaviour and ecology, both in terms of food preference and sexual behaviour. Because the composition of volatiles drastically changes during food ripening and fermentation, they can potentially provide different cues to different species. Among the distinctive ecological niches occupied by the multitude of *Drosophila* species, *D. nigrosparsa* is a habitat specialist, restricted to the European montane/alpine zone with a maximum density at about 2000 m above sea level, and most abundant at the timber line^22^. Its life history starts being revealed, and its preferred oviposition substrate are fungi^23^. Given the harsh conditions of the habitat, the fly likely evolved particular adaptations to inhabit the alpine ecosystem.

Here, as a first step to understand the genetic basis of the species’ ecological adaptation and life history, we analysed the genetic and the molecular architecture of the majority of genes related to chemosensation, aiming to identify signatures of evolutionary pressures (positive, purifying, and relaxed selection). For a wider view of the adaptation process for *D. nigrosparsa*, we combined genomics and structural biology. In doing so, after sequencing the *D. nigrosparsa* genome, and using transcriptomic data, we annotated the four main chemosensory gene families (CGFs), analysed their repertoire, and inferred structural changes related to the ligand binding domain of proteins under selection.

## Results

### CGF gene annotation

**Figure 1:**
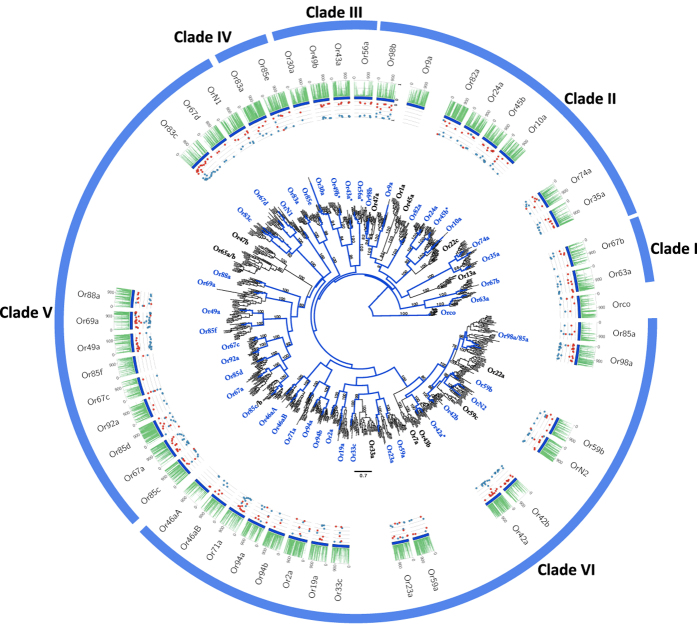
Phylogenetic relationships of the candidate *D. nigrosparsa* Odorant Receptors (ORs). Subdivided into six clades, the phylogeny describes *D. nigrosparsa* loci with respect to 13 other *Drosophila* species. The maximum likelihood tree was rooted by the OR co-receptor orthologs. Bootstrap support ≥70 for branches is indicated. Branches coloured in blue correspond to the lineages leading to *D. nigrosparsa* loci. Asterisks after the OR name indicate genes under positive selection. The histogram represents highly conserved elements (PhastCons) of each alignment. The PhastCons index ranges between 0 and 1 and can be interpreted as the probability that each base is a conserved element, based on the assumptions of the model and the maximum-likelihood parameter estimates. The dot plots report the distribution of synonymous (*α*; blue) and non-synonymous (*β*; red) substitution rates over sites inferred by the MEME model, which ranges between 0 and 1 (posterior probability >0.95).

To characterize adaptation in the CGF gene repertoire in *D. nigrosparsa*, we sequenced its genome (SI Appendix 1) and annotated CGF genes by mapping previously annotated *Drosophila* CGF protein sequences against the *de novo D. nigrosparsa* genome assembly. The analysis for ORs resulted in 55 candidate OR loci (SI Appendix 1, Fig. 1, Table S1), grouped in 45 of the 58 clusters obtained from the phylogenetic analysis of all *Drosophila* OR loci. The number of OR clusters did not differ from those in other *Drosophila* subgenus species and *S. flava* (minimum 43 in *D. grimshawi*; maximum 47 in *D. mojavensis*). Here, we define two terms: in-paralogs and out-paralogs. In-paralogs are paralogs in a given lineage produced by duplications after the split, which gave rise to that lineage and therefore represent lineage-specific gene expansions. Out-paralogs are paralogs in a lineage that evolved by gene duplications before emergence of the lineage^24^. This distinction is key to identifying lineage-specific gene expansions and adaptations. In nine clusters, we recovered paralogous genes, in seven of them only out-paralogs (*DnigOrN2, DnigOr42, DnigOr66d, DnigOr30a DnigOr98b, DnigOr10a, DnigOr74a*), in one only in-paralogs (*DnigOr9a*), and in another cluster one out-paralog and two in-paralogs (*DnigOr83c*, Fig. 1). Protein domain predictors found the *7tm*_6 Pfam domain in all loci, and a prediction from one to seven TMHs (Figure S2a). Because ORs bear 7-TMHs, for this study, we considered as putatively functional proteins those having: (i) a minimum of 6-TMHs, and ii) a Pfam domain coverage ≥70%^25^. A total of 33 loci were assigned as functional, while the remaining nine were classified as putative pseudogenes. We found that the distribution of TMHs predicted for the receptors in clade V, encompassing Or67d to Or85c (Fig. 1), was biased towards 6-TMHs instead of 7-TMHs. Namely, 89% of sequences were predicted with 6-TMHs, compared with 17% in the other clades. Looking at the domain sequence identities of this cluster, we observed a significantly lower value in clade V (average sequence identity of 22%) than in all other clades (average sequence identity of 25%; *p*-values < 0.002, one-tailed Wilcoxon rank-sum test; Figure S2b).

**Figure 2:**
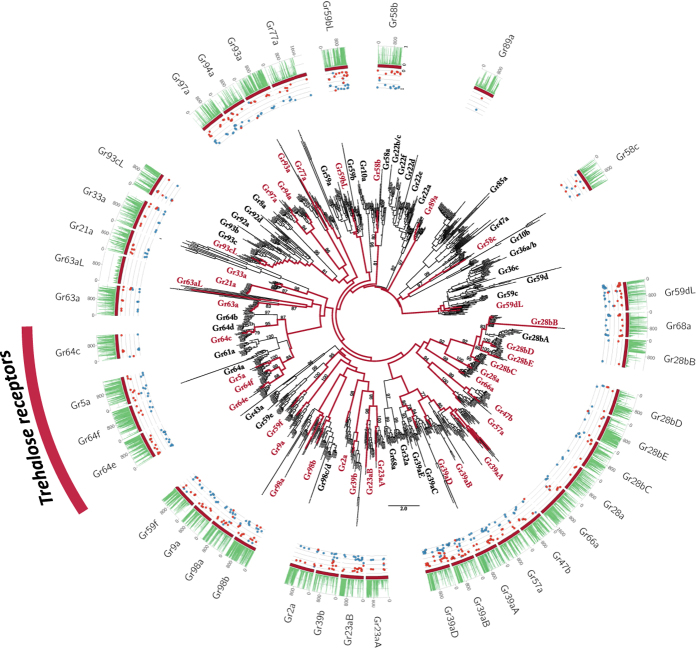
Phylogenetic relationships of the candidate *D. nigrosparsa* Gustatory Receptors (GRs). The phylogeny describes *D. nigrosparsa* loci with respect to 12 other *Drosophila* species. The sugar-related genes are highlighted as trehalose receptors. The maximum likelihood tree was midpoint rooted. Bootstrap support ≥70 for branches is indicated. Red branches correspond to the lineages leading to *D. nigrosparsa* loci. Asterisks after the GR name indicate genes under positive selection. Histogram and dot plots as in Fig. 1.

Forty-seven candidate loci for the GR gene family were annotated. All genes and transcripts were aligned to build the GR phylogenetic family tree. Of the 72 clusters, *D. nigrosparsa* GR loci grouped into 37, lacking between eight and 12 loci. Paralogs were found in five clusters, of which three contained just out-paralogs (*DnigGr59dL, DnigGr93cL*, and *DnigGr98a*), one only in-paralogs (*DnigGr58b*), and one three out- and two in-paralogs (SI Appendix 1, Fig. 2, Table S2). Protein domain predictors found the *7tm*_7 Pfam domain in 43 loci and the *Trehalose_recp* domain in four, and the protein topology predictions found between four and eight TMHs (Figure S2c). Applying the same criteria as for ORs (see above); 31 loci were assigned as functional and six as putative pseudogenes.

**Figure 3:**
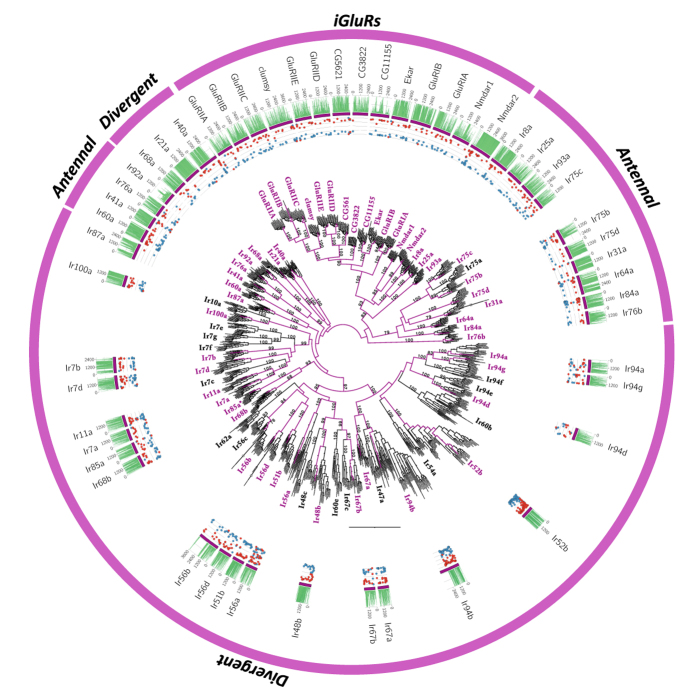
Phylogenetic relationships of the candidate *D. nigrosparsa* Ionotropic Receptors (IRs). Phylogenetic relationships of putative Ionotropic (IRs) and Ionotropic Glutamate receptors (iGluRs) in *D. nigrosparsa* and 12 other *Drosophila* species. IRs are subdivided into *antennal* and *divergent*. The maximum likelihood tree was midpoint rooted. Bootstrap support ≥70 for branches is indicated. Purple branches correspond to the lineages leading to *D. nigrosparsa* loci. Asterisks after the IR name indicate genes under positive selection. Histogram and dot plots as in Fig. 1.

About ionotropic receptors, we annotated both odour-specific IRs and iGluRs and found a total of 54 loci, clustering in 53 of the 67 clusters (SI Appendix 1, Fig. 3, Table S3). All 14 members of Kainate, AMPA, and NMDA receptors^5^ were found. We recovered all iGluRs, 17 of the 18 *antennal* and 21 of the ~30 *divergent* IRs. Only in-paralogs were found in one *antennal* (Ir75b) and in one *divergent* IR (Ir87a). Of the 39 loci, 26 (67%) were recovered with a complete gene model, of which only one was assigned as putative pseudogene (the *divergent* IR *DnigIr94d*). Protein domain predictors found *Lig_chan-Glu_bd* and *Lig_chan* domains, with a different degree of divergence among subfamilies. The iGluRs were recovered with sequence identities of 40% and 49% for *Lig_chan-Glu_bd* and *Lig_chan* domains, respectively. In contrast, between the other two IR subfamilies *antennal* and *divergent* divergence increased significantly. In detail, similarity of the *Lig_chan-Glu_bd* domain decreased from 24% in *antennal* to 18% in *divergent* and similarity of the *Lig_chan* domain decreased from 26% in *antennal* to 16% in *divergent (p*-adjusted <0.05, Wilcoxon rank-sum test; Figure S2d).

**Figure 4:**
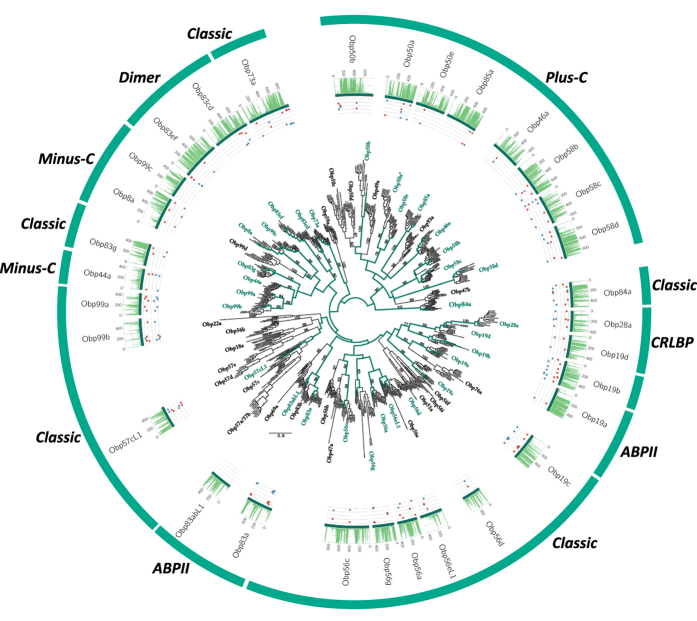
Phylogenetic relationships of the candidate *D. nigrosparsa* Odorant Binding Proteins (OBPs). The gene family tree describes phylogenetic relationships of the candidate *D. nigrosparsa* OBPs with the 12 other *Drosophila* species, subdivided according to the classic cysteine criteria. The maximum likelihood tree was midpoint rooted. Bootstrap support ≥70 for branches is indicated. Green branches correspond to the lineages leading to *D. nigrosparsa* loci. Asterisks after the OBP name indicate genes under positive selection. Histogram and dot plots as in Fig. 1.

Thirty-two candidate OBP loci were identified, aligned and grouped in 31 of the 57 *Drosophila* clusters (SI Appendix 1, Fig. 4, Table S4), 30 assigned as putative full-length OBPs, and between 10 and 11 loci were assigned as missing. Only one in-paralog was found, the *Plus-C DnigObp58b*, and based on conserved cysteines, one locus (*DnigObp19b*) was annotated as putative pseudogene, bearing only four of the six conserved cysteines. Protein domain predictors found the PBP_GOBP Pfam domain in all loci.

### Evolutionary forces on *D. nigrosparsa* chemosensory genes and their structural rearrangements

**Figure 5:**
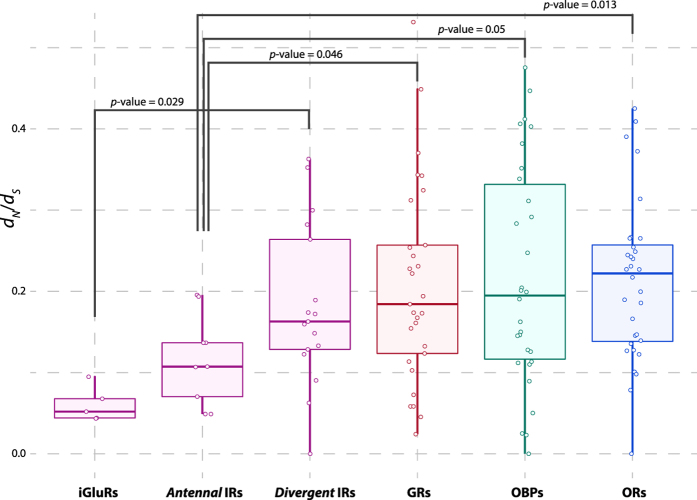
Evolutionary pressure in the *D. nigrosparsa* chemosensory gene families and sub-families. Boxplots depicting distribution and median (horizontal line) of *ω (d*_*N*_*/d*_*S*_) rates in each gene family and sub-family for *D. nigrosparsa*. Distributions were significantly different in four comparisons (Wilcoxon rank-sum tests).

After the CGF annotation, episodic diversifying selection was tested only on full-length loci. Putative signals of selection were recorded in five of 28 OR loci tested (18%; *DnigOr42a, DnigOr49ba, DnigOr43a, DnigOr56a, DnigOr45a*), in one of 24 GR loci tested (4%; *DnigGr64e*), in two of 21 odorant-related IRs tested (9%; *antennal DnigIr84a. divergent DnigIr85a*), and in one of the 29 OBP loci tested (3%; *Plus-C DnigObp50a*). The distribution of the mean *ω (d*_N_/*d*_S_) across the *D. nigrosparsa* branches indicated that the iGluRs and *antennal* IRs are evolving under significantly different selective pressures. The iGluRs (medians: *ω*_iGluR_ = 0.06) were found to be under stronger purifying selection than the IRs (*ω*_Divergent_ = 0.17; *p*-value = 0.028) and the *antennal* IRs (*ω*_Antennal_ = 0.12) under stronger purifying selection than the other three gene families (*ω*_Ors_ = 0.23, *p*-value = 0.013; *ω*_Grs_ = 0.19, *p*-value = 0.046; *ω*_Obps_ = 0.20, *p*-value = 0.051; one-tailed Wilcoxon rank-sum test) (Fig. 5). Although the ORs showed a higher rate of positive selection, the medians from ORs, GRs, and OBPs were statistically indistinguishable from each other (*p*-value > 0.05; one-tailed Wilcoxon rank-sum test). Given the generally stronger purifying selection in *antennal* IRs than the other gene families, we searched for a putative signal of relaxation amongst the full-length *antennal* IRs in *D. nigrosparsa*, and detected a significant relaxation rate (*k* < 1) in *DnigIr21a (ω* = 0.10; *k* = 0.78) and *DnigIr93a (ω* = 0.14; *k* = 0.67) (*p*-adjusted < 0.05) (Figure S3).

**Figure 6:**
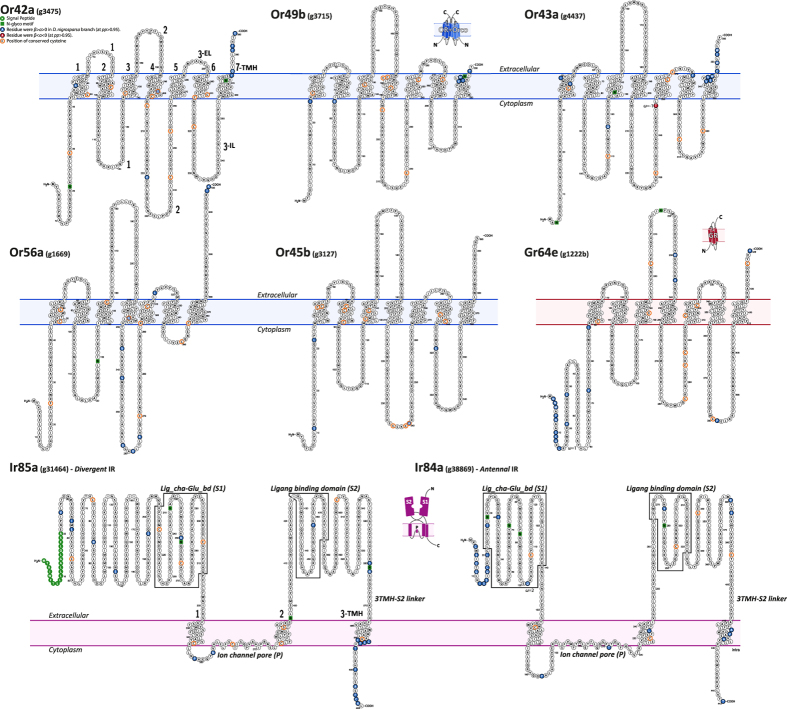
Protein topology of *D. nigrosparsa* receptors under positive selection. The predicted topologies of odorant, gustatory, and ionotropic receptors, showing distribution of non-synonymous substitutions (*β* > *α* > 0) on conserved residues (see methods, Figure S4) between *D. nigrosparsa* and the 12 *Drosophila* species (blue circles) and conserved cysteines (orange circles) along protein domains. Seven trans-membrane domains were inferred for ORs and GRs, three for IRs.

Given the absence of suitable OR/GR templates in Protein Data Bank, prediction of three-dimensional models would not be reliable^6^. Therefore, we focused on the prediction of the receptor topology organization. *Drosophila nigrosparsa* ORs had a general topology organization of 7-TMHs connected by three intracellular (ILs) and three extracellular loops (ELs) of variable lengths^6^. These receptors also included two variable regions at the N-terminus and C-terminus that protrude toward the cytosolic and extracellular compartments (Fig. 6)^6^. There are three critical regions for the functionality of these receptors: the N-terminus, the EL2, and IL3/TMH7, which have strong evolutionary constraints^6^,^26^. Performing site-to-site selection tests (MEME^27^) on loci under putative episodic positive selection, we aimed to identify codons for which non-synonymous (*β*) substitution rates are significantly higher than synonymous (*α*) substitution rates and zero (*β* > *α* > 0). The aim was to identify *D. nigrosparsa* branch-specific mutations with possible implications for protein structure functionality. We observed that a great portion, 48% (50/105) of all modifications, occurred on the amino and carboxyl terminus in all proteins, corresponding to the more variable and disordered protein regions^28^. The remaining 52% occurred on TMHs, ILs, and ELs. To better characterize the receptor structural rearrangements, we focused our analysis on highly conserved regions between *D. nigrosparsa* protein sequences and their orthologs^29^,^30^. Eight point mutations were isolated on ORs, which were grouped according to their structural localization: DnigOr42a_C196S_, (i.e. C196S represents a cysteine substituted by a serine in position 196), and DnigOr56a_C224G_ in TMH4; DnigOr42a_L221K_, DnigOr43a_T276F_, DnigOr56a_T258T_, DnigOr56a_E261R_, and DnigOr45b_C240A_ in IL2; and DnigOr45b_T319E_ in IL3. Two were identified in GR64e: DnigGr64e_L230W_ in EL2 and DnigGr64e_D381P_ in IL3 (Figs 6, S4).

**Figure 7:**
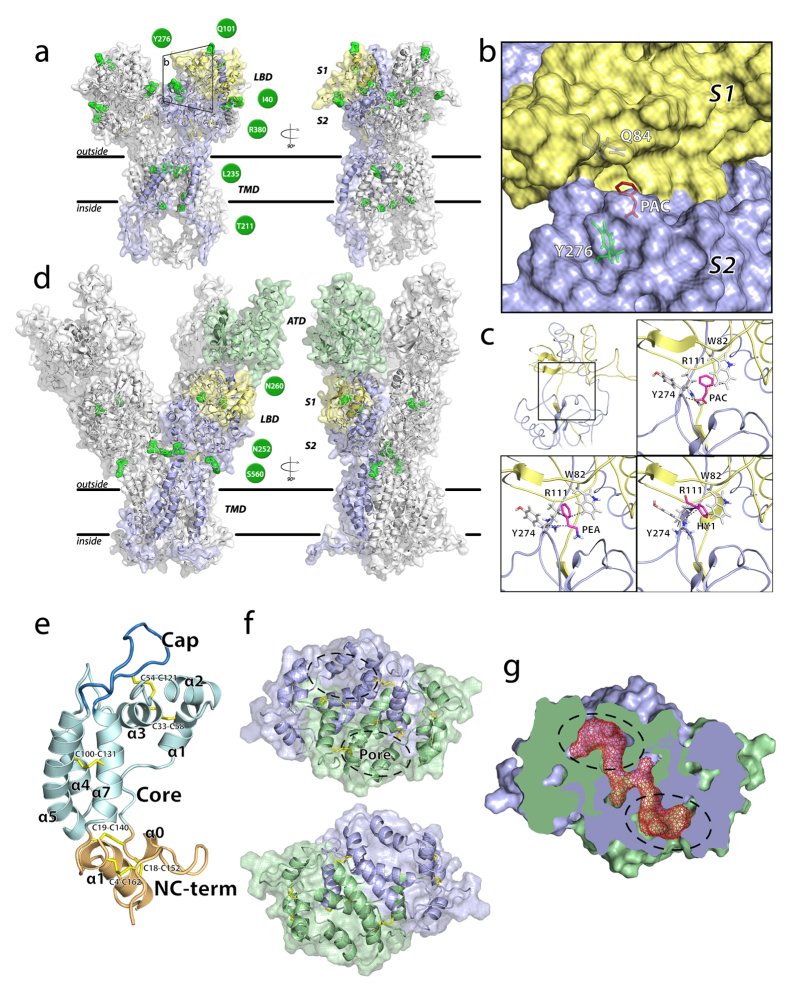
Three-dimensional protein structure prediction of DnigIr84a, DnigIr85a, and DnigObp50a. Protein models of DnigIr84a (**a**) and DnigIr85a (**d**) homotetramers. Only one chain was colour-coded according to its protein domains (yellow: *Lig_chan-Glu_bd*, Pfam PF10613; lilac: *Lig_chan*, Pfam PF00060; green: ATD, Pfam PF01094; TMD: transmembrane domain). The ligand binding domain (LBD) is subdivided in two halves, S1 and S2. Residues for which *β* > *α* > 0 in conserved regions (Figure S4) are depicted in green, as well as position and residue, which are shown in green circles. (**b**) Binding pocket of DnigIr84a. Its pore differs from *D. melanogaster* by two residues, Q84 and Y276. (**c**) The ligand-binding site of DnigIr84a and the three best pose for PAC, HY1, and PEA. (**e**) Schematic representation of the DnigObp50a and its three domains, the *Core* (pale green), the *Cap* (blue), and the *NC-term* domain (ochre). Sequence position of cysteines forming disulfide bonds are shown (yellow), together with *α*-helices. (**f**) Schematic representation of DnigObp50a three-dimensional domain-swapped dimer (lilac and green). Two views are shown, one from the face of the dimer formed by the swapped *NC-term* domain (up) and one from the face formed by the *α*5 and *α*5′ helices (bottom). A tunnel with pores on each side runs through the dimer interface. (**g**) Surface representation of the DnigObp50a swapped dimer (slice). The tunnel is represented by red mesh.

On the two IR receptors under positive selection (Fig. 7a,b), the site-to-site test helped us identify candidate structural changes. A total of 45 codons for the two loci were identified, most of them in variable and/or disordered regions, such as the C-terminus and the divergent ATD (i.e., *ANF_receptor*, Figure S2d)^31^, involved in channel assembly and/or co-factor binding^32^. Only nine mutations were located in conserved regions (Figure S4): three occurred in the *Lig_cha-Glu_bd* (S1) domain (DnigIr85a_H260N_, DnigIr85a_M401I_, DnigIr84a_G101Q_), three (DnigIr85a_H560G_, DnigIr85a_S562N_, DnigIr84a_Q380R_) in the TMH3-S2 linker, the region connecting S2 with TMH3, one (DnigIr84a_A221T_) in the ion channel pore (P), one (DnigIr84a_M235L_) in TMH2, and one (DnigIr84a_F276Y_) in S2 (Figs 6 and 7).

The homology between IRs and iGluRs allowed us to predict reliable three-dimensional structure models important to infer possible structural role of mutated residues in the binding site. We analysed the predicted structure of Ir84a and characterized it in terms of cavities and binding pockets. Two large cavities were located in the ion channel domain and in the region of Ir84a corresponding to the binding site of the iGluRs^33^,^34^, constituted by 28 residues. Using as reference DmelIr84a, we identified six mutated residues (21%; DnigIr84a_K81T_, DnigIr84a_E84Q_, DnigIr84a_D87P_, DnigIr84a_R101Q_, DnigIr84a_L114P_, DnigIr84a_F276Y_) and a proline deletion immediately after DnigIr84a_Y274_ (Fig. 7). All amino acids forming the putative binding site cavity were used as active residues (i.e., residues likely to be involved in binding) in three docking simulations, each using a specific small compound as ligand: phenylethylamine (PEA), phenylacetic acid (PAC), and phenylacetaldehyde (HY1)^17^. Complexes predicted by the HADDOCK algorithm were grouped in clusters according to the fraction of common contacts (fcc). PEA and PAC had higher average HADDOCK scores of the most representative cluster for the three ligands than HY1 (89%, 72%, and 23% for the refined complexes of HY1, PEA, and PAC, respectively). The PAC molecule had the best pose among all the ligands in terms of HADDOCK scores. Best scoring complexes for the DnigIr84a-ligand PEA and PAC suggested that three residues in the binding site (DnigIr84a_W82_, DnigIr84a_R111_, DnigIr84a_Y274_) might play a pivotal role in the binding of the three ligands. In details, these residues contacted the aromatic compounds in more than 90% of the proposed docking solutions. However, in more than 60% of the PEA poses, residue DnigIr84a_Q84_ established a hydrogen bond with the amino group of the PEA. Three other contacts were found in more than 90% of the predicted HY1 complexes: DnigIr84a_R111_, DnigIr84a_S273_, and DnigIr84a_R324_, which mainly established hydrophobic interactions.

A single OBP was detected with a signature of positive selection, *DnigObp50a*. While the translated putative protein sequence showed a good phylogenetic signal with other *Drosophila* orthologs (bs = 98), the locus was topologically basal and quite distant from other loci (Fig. 4), with a very low sequence similarity with respect to the loci from other species (between 47% and 62% with *D. erecta* and *D. mojavensis*, respectively). To understand the putative biological functionality, we built a three-dimensional model of *DnigObp50a*, and revealed a compact domain-swapped dimer structure (Fig. 7e,f). Each 193-amino-acid long monomer contained seven *α*-helices (*α*1-5, *α*7, *α*8 plus *α*0). The secondary structure element *α*6 was the only helix predicted with a random coil structure by HHpred^35^. The model had good structure similarity with the template (RMS: 2.644), and the monomer had the typical three-partition domain organization: a central *Core* domain, resembling the classical OBP fold (*α*1-7, and a loop); a flanking *NC-tem* domain, made by the two terminal domains linked by two disulfide bridges (*α*0 and *α*8); and a *Cap* domain made by the coil region and a long protruding loop. In contrast to the template, for which two channels are described, we found only a single L-shaped tunnel (Fig. 7g), bearing two pores formed by the equal contribution of the two monomers. As in the crystal structure, the inner cavity is located at the interface between *α*7 and *α*7′, which also represents one of the most conserved regions in the alignment. The conformation of the *NC-term* domain, which does not interact with the internal part of the *Core* domain, was oriented toward the solvent shaping a putative ligand binding site as already observed in other paralogs^19^,^36^.

### Putative odorant receptive landscape in D. nigrosparsa

**Figure 8:**
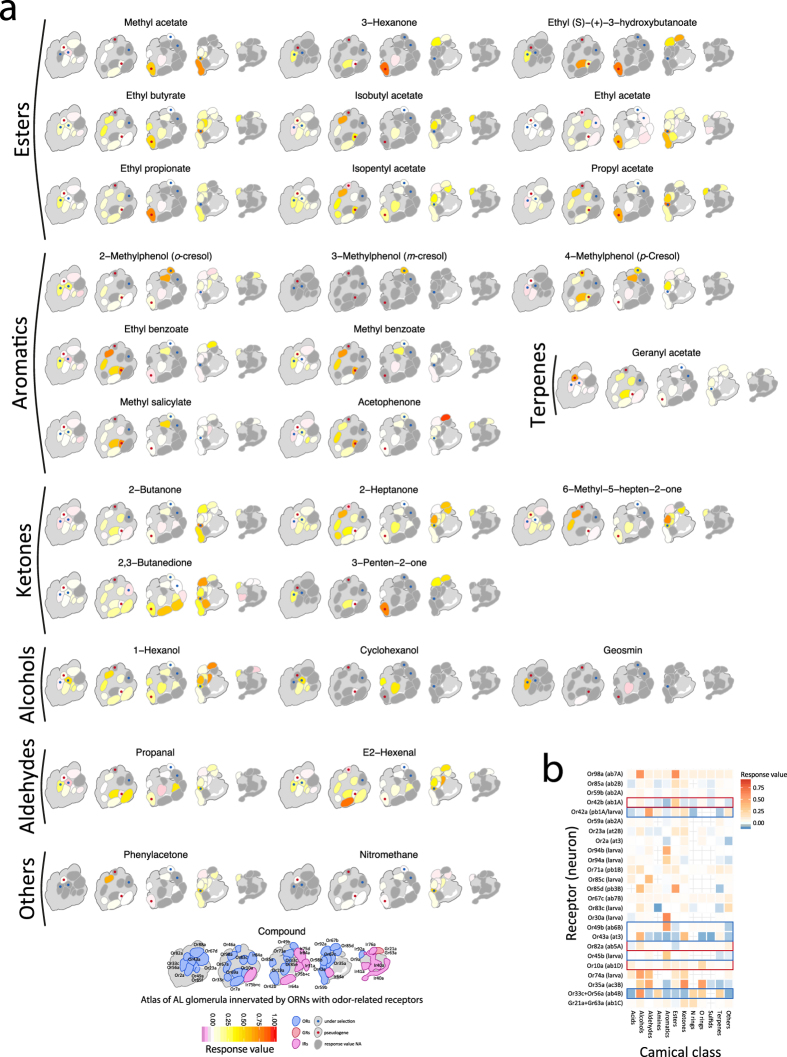
Odorant receptor-to-glomeruli projection map of response values to compounds and their chemical classes. In flies, like in other insects and vertebrates, odorants bind to odorant receptors of dendrites of bipolar olfactory receptor neurons (ORNs). Each ORN expresses one or very few receptors and sends its axons to make connections with the second order neurons in the glomeruli of the antennal lobes (ALs) of the brain. This figure shows (**a**) the heatmap response level of glomeruli of the AL connected with ORN expressing specific receptors with the more specific chemical compounds (response value ≥0.75) to the positively selected receptors as in *D. melanogaster*, whenever data are available; (**b**) the heatmap of response level of receptors with the different chemical classes. Blue and red rectangles represent pseudogenes and receptors under selection, respectively.

Once we had genetically characterized the putative chemosensory toolkit of *D. nigrosparsa*, we asked how this could reflect changes or modifications in the chemosensory receptive landscape of the species. Starting by looking at the available data of *D. melanogaster*’s olfactome and using the neural data from the Database of Odorant Receptors (DoOR v2.0.0)^37^,^38^, we mapped all available odorant interactions using the set of *D. nigrosparsa* receptors we characterized. Projecting knowledge on the peripheral and central aspects of odour detection of *D. melanogaster* on *D. nigrosparsa* may introduce errors, but lacking other options. We thus cautiously used the results of this approach to gain a first overview of the odorant reception landscape. Thereby, we showed that *D. nigrosparsa* possesses the full set of receptors capable of detecting a wide range of chemical classes (response value threshold ≥0.25) (Fig. 8). Focusing on odorant-related receptors under diversifying selection, we noted that Or42a, located both in the basiconic sensilla (palp) of the adult and in the dorsal organ dome in the larva of *D. melanogaster*^39^, might show a wide range of affinity to ~20 compounds including ketones, esters, aldehydes, alcohols, and others, all related to fruit scents and fermentation activity^14^,^39^. Interestingly, DnigOr67c had a significantly higher conservation level and was well affine to a wide range of chemical classes including its primary ligand, ethyl lactate, generated from malolactic fermentation and signalling a hospitable environment^14^. Or56a had a more specific pathway, a very narrow and conserved spectrum of action in *Drosophila*^40^, and is related to the detection and a subsequent repulsion response to geosmin^41^.

## Discussion

Until today, 26 genomes of drosophilid species have been published. Of them, only five (excluding *S. flava*) belong to the *Drosophila* subgenus, as does *D. nigrosparsa*. Sequenced genome sizes range from 121 Mbp in *D. busckii* to ~200 Mbp in *D. mojavensis* (194 Mbp), *D. grimshawi* (200 Mbp), and *D. virilis* (206 Mbp), to 254 Mbp in *D. albomicans*. Generally, eukaryote genome size reflects the genomic content in repeated sequences^42^. The *Drosophila* radiation is not an exception; transposable elements (TE) accumulation is a major factor of genome size variation, and both TE accumulation and genome size are fully explained by the phylogeny^43^. According to the CGF phylogenies now available, *D. nigrosparsa*’s phylogenetic position is within the *Drosophila* subgenus. When considering just OR-based phylogenies, it is most closely related to *D. grimshawi* and *S. flava*. This is in line with phylogenetic reconstructions using the complete mtDNA and single-copy ortholog genes (Cicconardi *et al. submitted*). Given that our size estimation for the *D. nigrosparsa* genome of 221.5 Mbp is consistent with those for other drosophilid species (~200 Mbp), we believe we have sequenced the great majority of the genome. This is also supported by the almost full list (94.5%) of BUSCO ortholog genes annotated and the rather high RNA-seq mappability of ~84%^44^.

The genome of *D. nigrosparsa* allowed us to begin the investigation of the unique characteristics and adaptations of this mountain fly. Here, we studied the main four CGFs, annotating 189 loci and recording an uneven proportion of presence/absence across CGFs. Indeed, while the set of ORs, iGluRs, and *antennal* IRs looks quite comprehensive, many genes belonging to GRs, OBPs, and *divergent* IRs seem to be missing. In interpreting the lack of these loci, we tend to reject a bias in assembling the genome as causal. Rather, it may represent gene loss. As shown in previous studies, gene turnover rate tends to vary across chemosensory gene families; ORs, OBPs, and *antennal* IRs have the least dynamic rates, with lower death rate (δ) values across different models (average δ_OR_ = 0.0017, δ_OBP_ = 0.0011, δ_Ant IR_ = 0.0003), while GRs and divergent IRs have higher dynamic rates and about 2-fold higher death rates (average δ_GR_ = 0.0023; δ_Div IR_ = 0.0026)^45^. Specialist species such as *D. sechellia*^16^,^45^, exclusively depending on *Morinda citrifolia* as a host plant, and *D. erecta*, depending on *Pandanus candelabrum*, have actually lost several GRs^45^. Although in our study this hypothesis was not fully validated, it is plausible that, especially for GRs and *divergent* IRs, a number of genes could have been lost through pseudogenisation during *D. nigrosparsa*’s early adaptation and rapidly deleted from the genome^16^,^46^,^47^. Unfortunately, it is very difficult to validate this theory, and strong evidence of profound gene loss in *D. nigrosparsa* is lacking.

Stronger signs of adaptation could, instead, be found in several genes bearing signs of evolutionary pressures. We found that nine of the 11 full-length CGFs are under putative diversifying positive selection (five ORs, one GR, one *antennal* IR, one *divergent* IR, one OBP), two *antennal* IRs bear a putative signal of relaxation, and in six loci putative pseudogenisation occurred (three single-copy ORs, two out-paralogous ORs, and one *divergent* IR). Here, based on the functional roles of some of these genes, we present hypotheses on how *D. nigrosparsa* might have adapted. Or49b and Or42a are known to play an important role in the attraction to aromatic and alcoholic compounds related to food^14^,^48^, while Or56a is pivotal in the detection of geosmin, a volatile toxic compound produced by a number of fungi^49^, bacteria^50^, and cyanobacteria^51^, which activates a conserved pathway among all *Drosophila* species, triggering a single class of sensory neurons and targeting the DA2 glomerulus (Fig. 8)^40^. In *D. nigrosparsa*, Or56a and Or42a lost two cysteines, highly conserved across their orthologues, both at TMH4 (Fig. 6). Cysteines play a key role in structurally related receptors by the formation of disulfide bonds^29^,^52^. Thus, the loss of disulfide bridges, particularly when found in combination with other surrounding cysteines (i.e., DnigOr42a_C196S_ surrounded by: DnigOr42a_C139_, DnigOr42a_C203_, DnigOr42a_C243_, DnigOr42a_C254_), might have an impact on the receptors’ folding in the cytoplasmic membrane^29^,^53^, and/or mediate affinity to compounds^54^. This might allow a higher degree of the receptor’s flexibility. We also found that the residues of intracellular loops (IL2 and IL3) evolve at a high rate. In these loops, the chemical/physical change due to a single amino-acid mutation might influence intracellular recruitment of other proteins once the receptor is bound to a ligand, influencing the signal transduction mechanism and modulating the receptor’s ability to recognize molecular partners^29^,^53^,^55^. These changes could affect the ion channel formation and functioning once the heterodimer is formed^6^. *Drosophila nigrosparsa* is a mushroom breeder (with more than 93% of eggs deposited on them in experiments) but not specialised on a single fungal taxon^23^. Modifications in *DnigOr56a* receptor may thus possibly reflect important adaptation towards different types of toxic compounds possibly present in fungi, and their recognition in non-suitable fungi species might inhibit positive chemotaxis, avoiding the oviposition^40^. Given that fungi are known to produce various volatile substances such as 1-octen-3-ol (called mushroom alcohol)^56^, *DnigOr56a* receptor modifications may alternatively be key to identifying suitable oviposition substrate using substances produced by fungi. This scenario would be in line with findings for *Drosophila* specialised on yeast^57^, such as *D. melanogaster, D. mojavensis*, and *D. suzukii*^58^,^59^.

We also found signs of diversifying positive selection in odorant-related IRs, specifically in one *antennal (DnigIr84a*) and one *divergent (DnigIr85a*) IR. In *D. melanogaster*, Ir84a effectively binds specific aromatic compounds, like PAC and PEA, commonly present on food sources, and the activation of the Ir84a pathway may promote male courtship in the presence of food, complementing the functions of pheromone receptors in regulating mate choice, thus explaining the widespread *Drosophila* behaviour to mate predominantly on their food^17^. Given the availability of crystal structures of phylogenetically and functionally related iGluRs^33^,^34^,^60^,^61^, it was possible to investigate the structural role of the modifications in these two *D. nigrosparsa* IRs. By predicting the three-dimensional homotetramer models for both receptors, and considering the strong purifying selection acting on *antennal* IRs (Fig. 5), it is likely that, among others, *DnigIr84a* successfully contributes to the adaptation to an environment where food is scarce or available only for a short time^62^. To support this hypothesis, we studied the binding site cavity and its surrounding and found that 21% of the residues are different between DnigIr84a and DmelIr84a, and two residues (DnigIr84a_E84_ and DnigIr84a_F276_) outline the cavity opening. This might cause difference in the compound accessibility in the pocket between the two proteins in ligand binding kinetics and affinity. The close proximity of the DmelIr84a insertion to the cavity entering might also underlie differences in ligand entering and recognition.

Overall, the results obtained by the data-driven docking are in accordance with the experimental data on binding affinity available for *D. melanogaster* reported from *in vivo* experiments^17^. The most representative contacts identified by the docking simulations do not involve the aforementioned residues, with the exception of the hydrogen bond between DnigIr84a_E84_ and PEA. These findings point to a more indirect effect caused by differences in the geometry and electrostatic potential of the two binding sites, underlying a fine-tuning adaptation mechanism for this particular class of receptors.

GRs are together with some other IRs involved in the gustatory response. In insects, the taste receptor system not only covers a wide ligand spectrum of sugars, bitter substances, and salts but also includes reception of pheromone and somatosensory stimulants^63^. In characterizing *D. nigrosparsa* GRs, we found duplication events, and putative *loss-of-function* in three other receptors. In *Drosophila*, the response to sugar is typically mediated by classes of sensory neurons mediated by eight GRs, namely Gr5a, Gr61a, and members of the Gr64a–f cluster^64^,^65^. In *D. nigrosparsa*, we were not able to annotate four of them (Gr61a, Gr64a, Gr64b, Gr64d), but one of them, *DnigGr64e*, specifically related to low-sugar stimuli and linked to glycerol, a by-product of yeast fermentation present in fermented fruit, was detected as the only GR bearing a signature of positive diversifying selection. In this receptor, we found modifications occurring on EL2, a protein region possibly involved in the binding site^6^. Both the possible *loss-of-function*, and the positive selection in this important receptor cluster may represent a process of adaptation towards low sugar content food.

Odorant and gustatory receptors are located in dendrite membranes embedded in lymph in contact with the air through pores in the sensilla’s cuticle^1^,^66^. Chemicals need to enter the aqueous lymph to interact with the odorant receptors, and because OBPs contain hydrophobic binding pockets, they play a key role in solubilizing hydrophobic odours^1^,^67^,^68^. Among them, only Obp50a, secreted by auxiliary cells of the antennal trichoid sensilla^69^, was found with a signature of diversifying selection, but no information is available about its compound affinity. By reconstructing the DnigObp50a three-dimensional structure, we made plausible all signs of putative functionality suggested by conserved features such as the disulfide bonds and the *α* helix configuration and specific characteristics such as the configuration of the channel pore, very different from other *Drosophila* orthologues. Although more evidence is needed, we suggest that this protein represents a possible additional adaptation/innovation of this alpine species.

In conclusion, insect chemosensory systems have emerged as prominent models in neuroscience. The investigation of their functions and mechanisms has revealed surprising answers to fundamental questions of how animals detect and process chemical stimuli and adapt to the environment. Using our comparative-genomics and structural-proteomics approach, we have described putative signatures of adaptation in the four CGFs in a habitat-specialist fly. We have detected a stronger adaptation of ORs compared with GRs, IRs, and OBPs. Like the majority of Drosophilidae, *D. nigrosparsa* also has the chemosensory arsenal for the exploration of fermentation-related food source, and it is possible to hypothesize that mutations on several receptors might enhance their affinity to compounds, mediating the evolution of more specialized food preferences in both adults and larvae.

## Methods

### Sequencing and genome assembly

The genome of *D. nigrosparsa* was sequenced from 150 pooled individuals of the iso-female line Iso12 kept at the University of Innsbruck, approximately 50 generations after its establishment. Six gDNA libraries were constructed, three with a short insert size (nominal size < 500 bp), consisting of one overlapped paired-end library (Ovl) of 100–300 nucleotide (nt) read length and two standard paired-end libraries of 100 and 130 nt lengths, and three mate-pair libraries with longer insert size (nominal sizes 2 kbp, 5 kbp, 10 kbp), outsourced at IGA Technology Services (http://www.igatechnology.com). The Ovl library was sequenced on Illumina MiSeq, all others on Illumina HiSeq2500. Raw reads were quality filtered using Trimmomatic v.0.32^70^ with a sliding window equal to 10% of the read length and a quality score of 10 without trimming, deduplicated with FastUniq^71^, and error corrected with SOAPec^72^ (kmer = 17). The estimated raw base error ratio was very low at 0.0010 to 0.0003.

ALLPATHS-LG^73^ was used to assemble the genome, which was done in two iterations. In the first iteration, all reads were used to run a first draft genome (PLOIDITY = 2), using the Blobology pipeline^74^ to filter out possible contaminants. In brief, the pipeline consists of a first BLASTn v.2.2.29+ search of contigs against the whole nucleotide database of GenBank, retaining taxonomic information from the best hit, followed by mapping of genomic read libraries against contigs. By plotting each contig’s GC content versus coverage, possible contaminant contigs from organisms hosted by the target species were detected. All reads mapping to contaminant contigs were filtered out from each library and all remaining ones used to run the second iteration to assemble the genome. Genome completeness was assessed searching the 2675 arthropod orthologous genes from the Benchmarking Universal Single-Copy Orthologs (BUSCO)^75^ on a provisional complete genome annotation. Annotation of the genome was performed mapping all *D. melanogaster* protein sequences and RNA reads onto the genome and used as hints for the BRAKER pipeline^76^.

### Gene family datasets and identification of CGFs in Drosophila nigrosparsa

The dataset for all gene families from the *Drosophila* 12 genomes consortium^77^ was obtained from Almeida *et al*.^45^. For each gene family, both nt and amino acid (aa) sequences were used, together with the assignment of orthology. For ORs, both nt and aa sequences from *Scaptomyza flava*^78^ were added to the study.

To identify and characterize chemosensory genes in *D. nigrosparsa,* aa sequences from all available Drosophilidae species were used as queries in a tBLASTn v.2.2.29+ search on the *D. nigrosparsa* assembly with an *e*-value cutoff of 1e^−5 ^78. From all hits, genomic regions of 15 kbp were extracted from the assembly and used as input file for Augustus v.3.2.1^79^ (–genemodel = partial –species = fly). The *ab initio* transcriptome of *D. nigrosparsa* was assembled using TopHat^80^ and Cufflinks v.2.2.1^81^, using the available RNA-seq library of *D. nigrosparsa*^82^. Each gene was then manually annotated taking care of tBLASTn and Augustus results, together with the *ab initio* transcriptome and spliced reads. *Drosophila nigrosparsa* sequences that did not align to the entire length of orthologous *Drosophila* sequences were labelled incomplete only when gaps where present; sequences with nonsense mutations were annotated as putative pseudogenes. Gene annotation on the *D. nigrosparsa* genome was confined to the four gene families for this study.

### Phylogenies of chemosensory gene families and orthology assignment

We inferred the evolutionary relationships of the ORs, GRs, IRs, and OBPs annotated from the *D. nigrosparsa* genome, and 12 (13 for ORs) Drosophilidae species using aa sequences. Pseudogenes and incomplete genes were included in the analysis. For each CGF, aa sequences were aligned using ClustalW v.1.2.1 EBI web server^83^ (settings: mbed: true; mbediteration: true; iterations: 5; gtiterations: 5; hmmiterations: 5), and the phylogeny was inferred using maximum likelihood (ML) search as implemented in RAxML v.8.1.20^84^ (Rapid BS search with MRE-based bootstopping criterion “–f a”), using the best-fit amino acid substitution model inferred with ProtTest v.3.4.1^85^. For OR phylogeny, the Odorant receptor coreceptor (Orco/Or83b) was used as outgroup^78^,^86^. Gene orthology was subsequently assigned based on the phylogenetic tree.

To assign orthology, count the actual number of genes, evaluate functional completeness in each of the four protein families, and evaluate the presence or absence of genes in the genomes of *D. nigrosparsa* and the other species, we used the gene family phylogeny, manually annotated clusters of closely related genes, and adopted the classification by Sonnhammera & Kooninb^24^,^87^. Protein domains were searched using the HHpred server^35^, using both Pfam databases available, pfamA_29.0 and pfam_03Feb16.

### Gene nomenclature

Among the four datasets, only the IR family required a major curation given its lack of orthologous assignment (see previous section) and correspondence between aa and nt sequences. We manually curated the conformity and annotation by searching between annotated nt and aa sequences using BLAT. All *D. nigrosparsa* genes were named according to their orthology with *D. melanogaster*.

### Diversifying positive selection analysis

To assess roles of diversifying positive selection on the evolution of CGFs, signatures of selection were searched for each putative full-length gene of the four protein families. CDSs were aligned using MACSE v.1.01b^88^, which implements a pairwise CDS alignment method that detects CDS preserving codon structure. From each gene alignment, gaps and ambiguously aligned sites were removed with Gblocks v.0.91b^89^ (settings: −t = c −b1 = $b −b2 = $b −b3 = 1 −b4 = 6 −b5 = h; where $b is the number of sequences divided by two plus one) (Cicconardi *et al. submitted*). The alignment was afterwards used to infer a *de novo* gene tree with an ML search performed with FastTree v.2.1.8 SSE3^90^. Signatures of diversifying selection were inferred using the adaptive branch-site random effects likelihood (aBSREL^91^) algorithm as implemented in the HYPHY batch language^92^, testing only branches leading to *D. nigrosparsa* genes. Using the Benjamini and Hochberg method^93^, *p*-values were adjusted and a threshold of 0.05 used.

We performed the RELAX^94^ test to search for a putative signal of relaxation amongst the full-length *antennal* IRs in *D. nigrosparsa,* using as reference all branches. All *p*-values were adjusted for multiple comparisons. In brief, RELAX tests the hypothesis of evolutionary rate relaxation in selected branches of a phylogenetic tree compared with reference branches. A *k* value is computed to evaluate whether selective strength was relaxed (*k* < 1) or intensified (*k* > 1).

To detect site-wise positive selection, the mixed effects model of evolution (MEME)^27^ was used as implemented in HYPHY, searching for the best substitution model also implemented in HYPHY. The analysis was performed in an explorative manner, gaining insight into the proportion of negative and positive pressure for all genes (plots in Figs 1, 2, 3 and 4) using as threshold a posterior probability >0.95.

In an explorative analysis, highly conserved elements (HCEs) were also searched for each gene using the PhastCons algorithm^95^. This was done running PhyloFit (PHAST package v.1.3^96^) to estimate a neutral phylogenetic model (also considered as the nonconserved model in PhastCons), using as topology the one previously computed as the guide tree, followed by PhastCons (PHAST package) to estimate conservation for each nucleotide position of all aligned genes.

### Protein topology, modelling, and docking

To predict receptor transmembrane topology for all ORs, GRs, and IRs, the TOPCONS webserver v.2.0^97^ was adopted. Protein topology analyses and modelling were performed only for putative full-length proteins for which signs of positive selection were found. We were not able to predict the three-dimensional structure of ORs and GRs because no experimental structures with a satisfying amino acid sequence identity were available. We used the Protter webserver^98^ together with the HMMTOP webserver^99^ to draw the expected 7-TMHs topology.

Modeller v.9.16^100^ was adopted to predict three-dimensional structures of IRs and OBP. Two templates were used for DnigIr85a (pdb codes: 4u2p,4uqq), and another one for both DnigIr84a (pdb code: 4u2p) and DnigObp50a (pdb code: 4ij7). The homotetramers for IRs and the homodimer for DnigObp50a were obtained superimposing our models to AMPA subtype iGluR (pdb code: 3KG2).

The ligand-receptor complexes of DnigIr84a bound to three of its known ligands: PEA, HY1, and PAC^17^ were predicted using the HADDOCK v.2.2 algorithm^101^. The algorithm options were set as recommended for small molecule docking in Sennhauser *et al*.^102^, with final docking poses set to 500. Complexes were grouped in clusters according to the fraction of common contacts (fcc). We used the 3 V tool^103^ to calculate cavities on the three-dimensional model of Ir84a and highlight the residues shaping the putative binding site cavity. To analyze the resulting docking poses, we calculated all contacts under 4.0 Å distance between DnigIr84a residues and the small molecules using the R package Bio3d v.2.2-4^104^. Specific interactions for the most representative contacts were calculated using Ligplus^105^.

## Additional Information

**How to cite this article:** Cicconardi, F. *et al*. Chemosensory adaptations of the mountain fly *Drosophila nigrosparsa* (Insecta: Diptera) through genomics’ and structural biology’s lenses. *Sci. Rep.* **7**, 43770; doi: 10.1038/srep43770 (2017).

**Publisher's note:** Springer Nature remains neutral with regard to jurisdictional claims in published maps and institutional affiliations.

## Supplementary Material

Supplementary Information

Supplementary dataset 1

Supplementary dataset 2

Supplementary dataset 3
