# Supplementary material for: Chemosensory adaptations of the mountain fly Drosophila nigrosparsa (Insecta: Diptera) through genomics’ and structural biology’s lenses
Source: Sci Rep. 2017 Mar 3;7:43770. doi: 10.1038/srep43770 (PMC5335605; doi:10.1038/srep43770)
Supplement: Supplementary Information [file srep43770-s1.pdf]

Chemosensory adaptations of the mountain fly *Drosophila nigrosparsa* (Insecta: Diptera) through genomics' and structural biology's lenses

Francesco Cicconardi[1,*], Daniele Di Marino[2], Pier Paolo Olimpieri[3], Wolfgang Arthofer[1], Birgit C. Schlick-Steiner[1], Florian M. Steiner[1]

[1]Institute of Ecology, University of Innsbruck, Technikerstr. 25, 6020 Innsbruck, Austria.
[2]Department of Informatics, Institute of Computational Science, University of Italian Switzerland, Lugano, Switzerland.
[3]Department of Physics, Sapienza University of Rome, Rome, Italy.

Supplementary Information

Appendix1: Extended results

Genome sequencing. After filtering out contaminants and low quality reads, a final set of 0.6 terabases (Tb) of Illumina reads remained. Read number ranged from more than 10 millions (M) for overlapped reads (Ovl) to more than 230 M for paired-end (PE) reads (Figure S1). Based on an estimated genome size of 221.5 Mbp (Kmer spectrum; K=24), 80-fold coverage was used to assemble the genome, which had a coverage of 38.1 fold for Ovl reads, and ranged from 11.3 fold (10k mate-pairs (MP)) to 178.2-fold (second paired-end library (PE2)). The assembly was 116 Mbp long (168 Mbp including gaps), with an N50 value of 4.4 kb (35 k contigs; min size 1000 bp), a scaffold N50 of 263 kb (335 kb including gaps), and a GC level of 40%. The genome fraction with a copy number > 2 was estimated to be 18%, the length of the genome involved in repeats (e.g. retroelements and transposons) to be 19 Mbp (~12%). The number of observed single nucleotide polymorphisms (SNPs) and indels was 382 k (rate: 1/311; estimate rate: 1/89) and 145 k (rate: 1/821), respectively. The annotation pipeline gave a total of 13,484 loci, and 2527 of the 2675 (94.5%) arthropod BUSCO genes were found. Of them, 84.9% (2271) were recovered as complete and 9.6% as fragmented (C:84.9%[D:16.9%],F:9.6%,M:5.5%,n:2675). Raw reads

from previously sequenced RNA[1] gave extensive mapping results, with 118 million fragments (84.1%) correctly aligned.

Identification of candidate odorant receptors. The analysis of the *D. nigrosparsa* transcriptome and genome resulted in 55 candidate OR loci. These loci were aligned with all available *Drosophila* ORs[2] and with the recently added *Scaptomyza flava* ORs[3]. The phylogenetic family tree grouped all genes into 58 clusters. *Drosophila nigrosparsa* OR loci were present in 45 of them. Forty-one of these clusters were recovered with good bootstrap support (bs ≥ 75) (Figure 1). The number of OR clusters did not differ from those in other *Drosophila* subgenus species and *S. flava* (minimum 43 in *D. grimshawi*; maximum 47 in *D. mojavensis*). In nine clusters, we recovered paralogous genes, in seven of them only out-paralogs (*DnigOrN2*, *DnigOr42*, *DnigOr66d*, *DnigOr30a DnigOr98b*, *DnigOr10a*, *DnigOr74a*), in one only in-paralogs (*DnigOr9a*), and in another cluster one out-paralog and two in-paralogs (*DnigOr83c*, Figure 1).

Based on gapped regions flanking or overlapping exons, we distinguished between partial and putative full-length loci. Of the 55 loci, 42 (76%) were recovered with a complete gene model. Putative full-length *D. nigrosparsa* ORs were quite divergent, sharing between 44% (DnigOr85a) and 97% (DnigOr30a) of protein identity with their closest related amino acid sequence (average 70%). Greater sequence identity was observed in *S. flava* and *D. virilis* than in the other species. With these two, protein domain predictors found the *7tm_*6 PFAM domain in all loci, and a more detailed analysis on the putative full-length genes resulted in predicting from one to seven TMHs (Figure S2a). Thirty-three loci were assigned as functional, while the remaining nine were assigned as putative pseudogenes. The proportion of amino acid residue aligned to the PFAM protein domain varied between 82% and 100%, with a mean of 96%. Taking into account only full-length loci, we found that receptors in clade V, encompassing Or67d to Or85c (Figure 1), had a distribution of predicted TMHs with a bias towards 6-TMHs instead of 7-TMHs. Namely, 89% of sequences were predicted with 6-TMHs, compared with 17% in the other clades. Looking at the domain identities of this cluster, we observed a significant lower value in clade V

(average identity of 22%) than in all other clades (average identity of 25%; $p<0.002$, Wilcoxon rank-sum test; Figure S2b).

Identification of candidate gustatory receptors. Forty-seven candidate loci for the GR gene family were annotated. Based on read splicing and gene structure, three of these putatively transcribe into different splice variants: two loci with two isoforms, and one with four (Table S2). All genes and transcripts were aligned to build the GR phylogenetic family tree. Of the 72 clusters, *D. nigrosparsa* GR loci grouped into 37, of which 26 (70%) were recovered with good node support (bs ≥ 75) (Figure 2). Considering the number of clusters in the other three *Drosophila* subgenus species (45 for *D. grimshawi* and 49 for *D. virilis* and *D. mojavensis*), *D. nigrosparsa* with its 37 clusters seemed to lack between eight and 12 loci. Paralogs were found in five clusters, of which three contained just out-paralogs (*DnigGr59dL*, *DnigGr93cL*, and *DnigGr98a*), one only in-paralogs (*DnigGr58b*), and one three out- and two in-paralogs.

Of the 47 protein sequences, 37 (79%) were recovered with a complete gene model. Protein domain predictors found the *7tm*_7 PFAM domain in 43 loci and the *Trehalose_recp* domain in four. The protein topology predictions detected between four and eight TMHs (Figure S2c). Applying the same criteria used for ORs (see above), 31 loci were assigned as functional, while six were marked as putative pseudogenes. The average proportion of residues aligned to the two protein domains was 94%. Protein domain identities were, on average, 21% in the *7tm_7* domain and 44% in the *Trehalose_recp* domain (Figure S2d).

Looking at the full-length protein sequence diversity, sequence identities among *D. nigrosparsa* GRs and other *Drosophila* subgenera species have a slightly wider distribution, varying between 53% (DnigGr39aA) and 97% (DnigGr29bB), with an average identity of 76%. The highest sequence identity value was observed with *D. virilis* at 21 best hits, followed by *D. grimshawi* at 11 best hits.

Identification of candidate ionotropic receptors. We annotated both subfamilies and found a total of 54 loci. The phylogenetic family tree grouped all genes into 67 clusters (Figure 3). All 14 members of the three iGluR subfamilies (ten Kainate, two AMPA, and two NMDA receptors)[7] were found. The remaining

53 clusters were assigned to IRs, which are subdivided into two subgroups, *antennal* and *divergent* IRs[8]. The 39 *D. nigrosparsa* IR loci clustered with 38 IRs, 36 of which were recovered with good node support (bs ≥ 75) (Figure 3). We recovered 17 of the 18 *antennal* and 21 *divergent* IRs. While the number of *antennal* IRs is conserved within *Drosophila*, the *divergent* IRs do not show a similar conservation, ranging between 31 and 34 loci in the subgenus. This indicates a possible lack of genes, between 10 and 13 *divergent* IRs. Almost all *D. nigrosparsa* loci clustered as single copy genes, only for three genes two in-paralogs per cluster were found (CG11155, Ir75b, Ir87a; Figure 3). Of the 39 loci, 26 (67%) were recovered with a complete gene model, of which only one was assigned as putative pseudogene (*DnigIr94d*). We observed a significantly decreasing sequence similarity from iGluRs (median identity of the best hit: 91%) to *antennal* (81%) to *divergent* IRs (71%) (*p-values* < 0.001, Wilcoxon rank-sum test).

A different degree of divergence among subfamilies was observed for *Lig_chan-Glu_bd* and *Lig_chan* domains, which shared a sequence identity of 40% and 49% in their iGluR, respectively. In contrast, between the other two IR subfamilies *antennal* and *divergent* divergence increased significantly. In detail, similarity of the *Lig_chan-Glu_bd* domain decreased from 24% in *antennal* to 18% in *divergent* and similarity of the *Lig_chan* domain decreased from 26% in *antennal* to 16% in *divergent* (*p*-adjusted < 0.05, Wilcoxon rank-sum test; Figure S2d).

Identification of candidate odorant binding proteins. Thirty-two candidate OBP loci were identified and aligned to compute the phylogenetic family tree, which grouped all genes into 57 clusters. *Drosophila nigrosparsa* OBP loci clustered in 31 of them, with two in-paralogs in the Obp58b cluster. Twenty-four of these clusters (77%) were recovered as monophyletic with significant node support (bs ≥ 75), one as paraphyletic although not supported (Obp19d), and six as monophyletic without statistical support (Figure 4). There were 44 gene clusters present in the *Drosophila* subgenus species, ranging between 41 and 42 in the different species; and between ten and 11 loci were missing in our annotation (Figure 4).

Putative full-length OBPs were recovered for 30 of the 32 annotated loci. OBPs were less diverging than other chemoreceptor families, sharing between 32% (DnigObp50b) and 93% (DnigObp44a) of their closer amino acid sequence (average identity of best hits: 78%). Higher values of sequence identity were observed in *D. virilis*. Protein domain predictors found the PBP_GOBP PFAM domain in all loci, and a more detailed analysis of the conserved cysteines revealed one putative pseudogene of *DnigObp19b,* bearing four of the typically six cysteines. The proportion of amino acid residue aligned to the protein domain varied between 72% and 100%, with a mean of 93%, and 23 of 31 loci aligned more than 90%. Domain identities varied considerably, ranging from 13% in the duplex *Obp83ef* to 39% in *Obp56d*, with a mean identity of 24% (Figure S2d).

References

1. Arthofer, W. *et al.* Genomic Resources Notes Accepted 1 August 2014 – 30 September 2014. *Mol. Ecol. Resour.* **15,** 228–229 (2014).
2. Almeida, F. C., Sánchez-Gracia, A., Campos, J. L. & Rozas, J. Family size evolution in *Drosophila* chemosensory gene families: a comparative analysis with a critical appraisal of methods. *Genome Biol. Evol.* **6,** 1669–82 (2014).
3. Goldman-Huertas, B. *et al.* Evolution of herbivory in Drosophilidae linked to loss of behaviors, antennal responses, odorant receptors, and ancestral diet. *Proc. Natl. Acad. Sci.* **112,** 201424656 (2015).
4. Joseph, R. M. & Carlson, J. R. *Drosophila* chemoreceptors : a molecular interface between the chemical world and the brain. *Trends Genet.* **31,** 1–13 (2015).
5. Hopf, T. A. *et al.* Amino acid coevolution reveals three-dimensional structure and functional domains of insect odorant receptors. *Nat Commun* **6,** 6077 (2015).
6. Leoni, G., Le Pera, L., Ferrè, F., Raimondo, D. & Tramontano, A. Coding potential of the products of alternative splicing in human. *Genome Biol.* **12,** R9 (2011).
7. Benton, R., Vannice, K. S., Gomez-Diaz, C. & Vosshall, L. B. Variant ionotropic glutamate receptors as chemosensory receptors in *Drosophila*. *Cell* **136,** 149–162 (2009).
8. Croset, V. *et al.* Ancient protostome origin of chemosensory ionotropic glutamate receptors and the evolution of insect taste and olfaction. *PLoS Genet.* **6,** e1001064 (2010).

Appendix 2: Supplementary figures

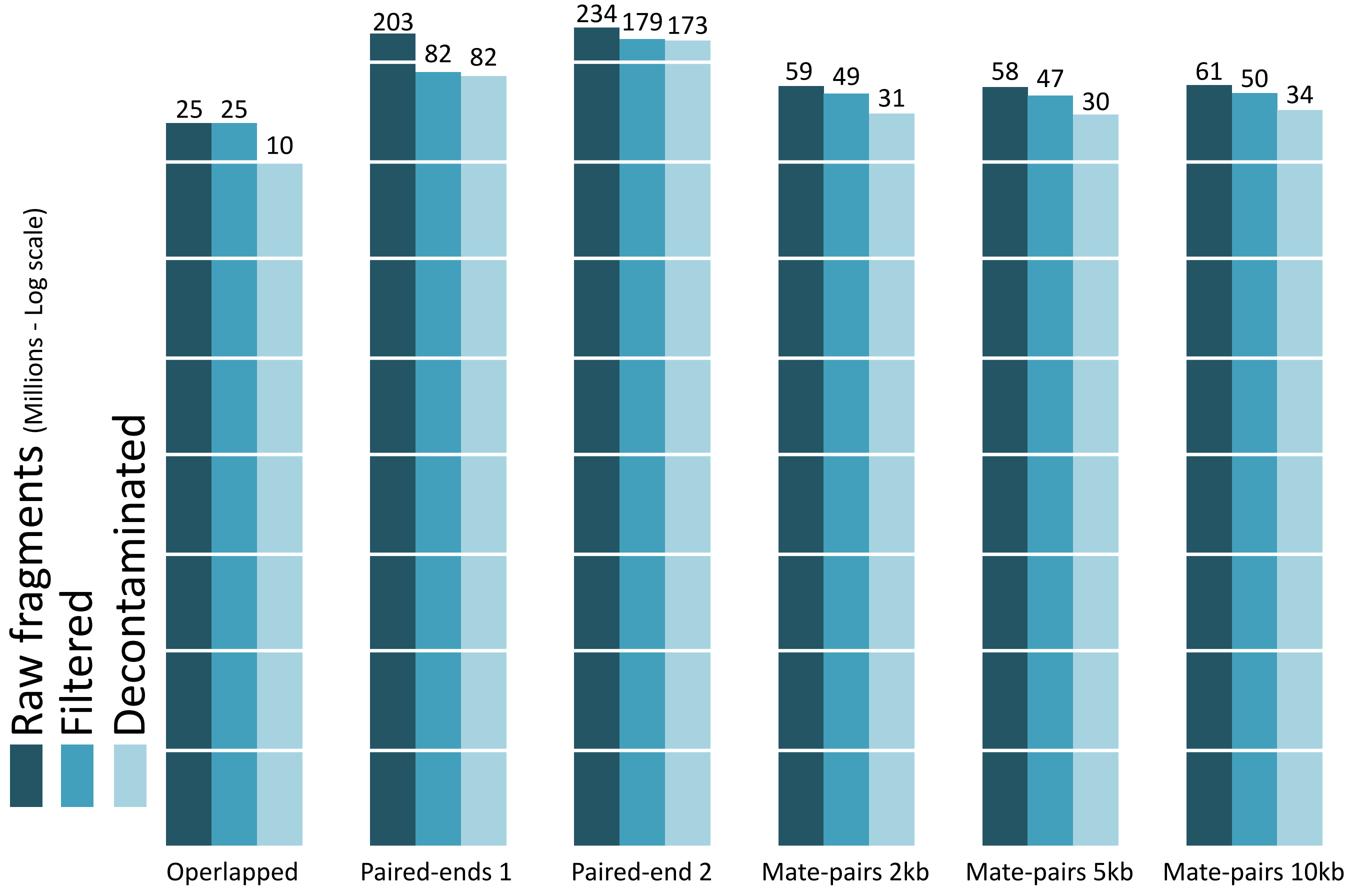

Figure S1 – Sequenced read abundance for each genomic library. Log-scale histograms of the abundance of raw, filtered, and decontaminated reads for each library.

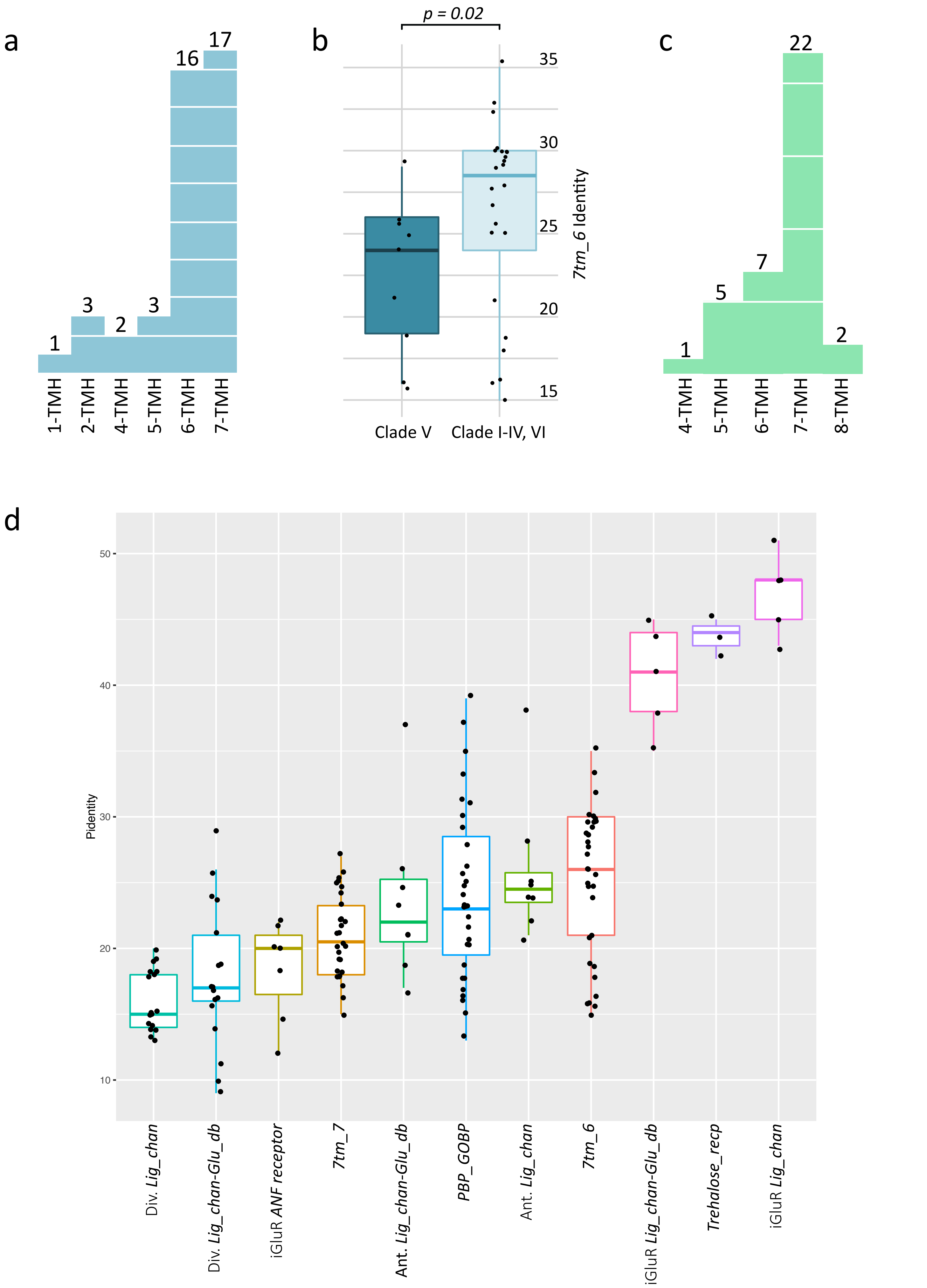

Figure S2 – Transmembrane helix prediction and sequence identity of the CGFs. Histograms describe the distribution of the predicted number of TMHs for each odorant (a) and gustatory (c) receptor loci. b) The two boxplots show the significantly different distribution of *7tm_6* domain (PFAM PF02949) protein identities between Clade V and Clades I-IV, VI (Wilcoxon rank-sum tests). d) Protein identity distribution of all conserved PFAM domains (colour-coded; Div: *divergent* IRs; Ant. *antennal* IRs).

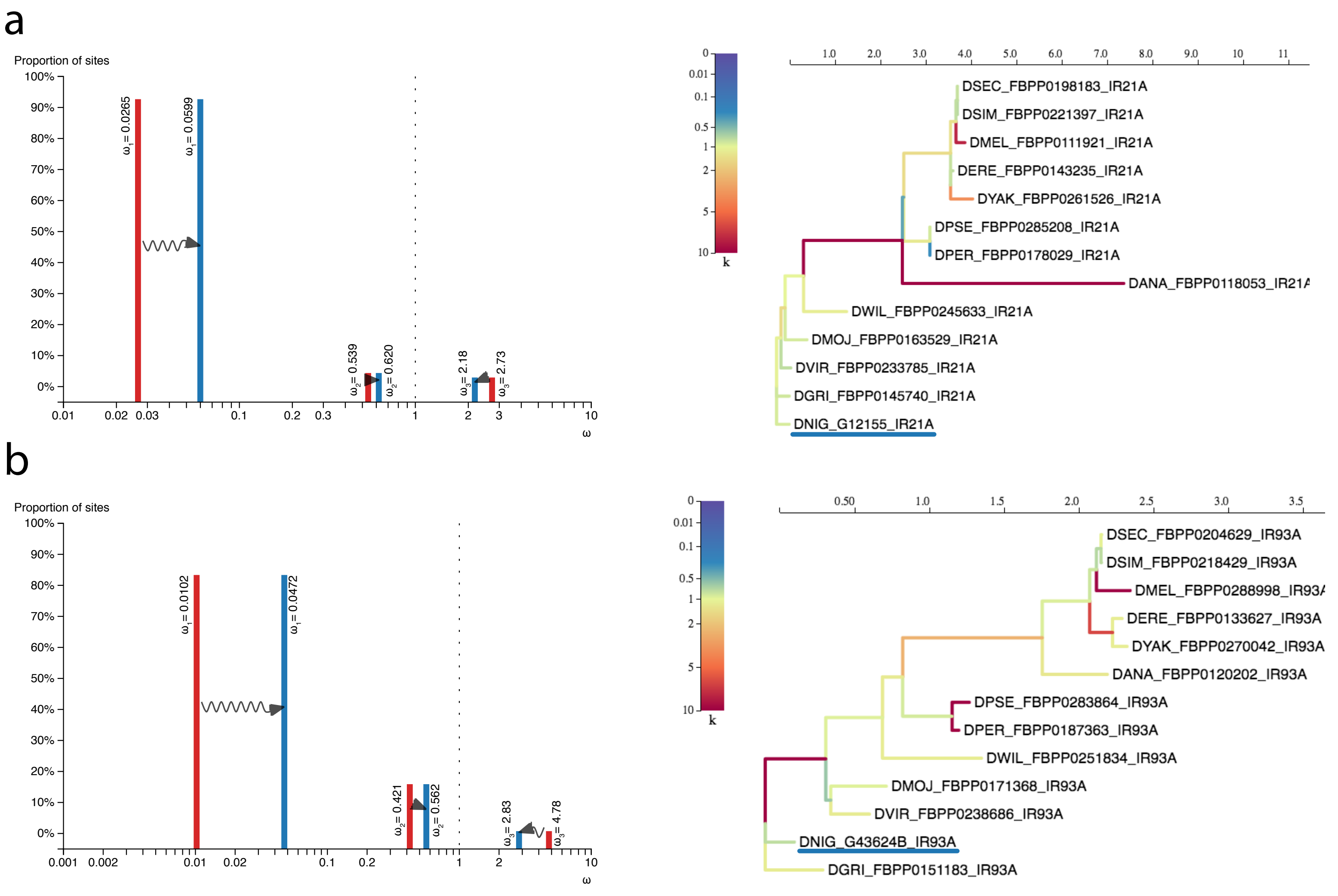

Figure S3 – Pattern of relaxed purifying selection in *antennal D. nigrosparsa* IRs. Selection profiles in a) *DnigIr21a* and b) *DnigIr93a*. Three *ω* parameters and the relative proportion of sites are plotted for the tested *D. nigrosparsa* branch (blue) and the reference (red) branches, the whole gene family phylogeny. Only *ω* categories representing non-zero proportions of sites are shown. Arrows show the shift of tested branches with respect to reference branches. Unrooted gene trees showing branch specific parameters inferred for the two receptors under the General Descriptive model. Branches are coloured based on relative value of selection intensity parameter *k*.

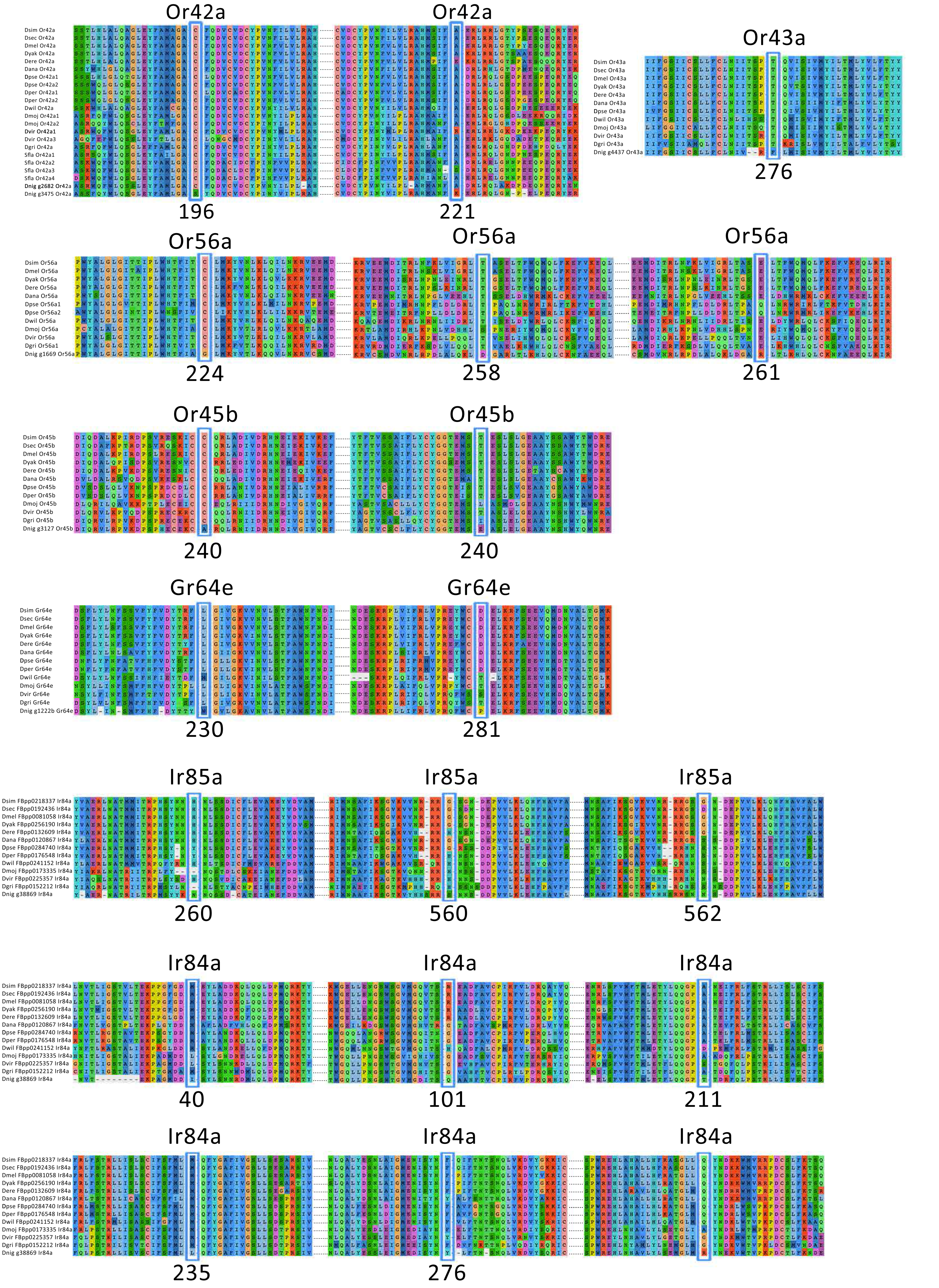

Figure S4 – Colour-coded local alignment of non-synonymous substitutions ($\beta > \alpha > 0$).
For each selected non-synonymous substitution in all loci under positive selection, its sequence position and flanking region were locally aligned to show the conservation level.
